# OLDER ADULT CAREGIVERS’ THOUGHTS ON WOUND CARE RESOURCES

**DOI:** 10.1093/geroni/igac059.3113

**Published:** 2022-12-20

**Authors:** Kristen Swartzell, Janet Fulton, Jane Von Gaudecker

**Affiliations:** IU School of Medicine, Indianapolis, Indiana, United States; Indiana University, Indianapolis, Indiana, United States; Indiana University, Indianapolis, Indiana, United States

## Abstract

As healthcare increasingly shifts to home and community-based settings, informal caregiver responsibilities are increasing beyond assistance with activities of daily living to include complex care procedures previously performed by licensed caregivers in clinical settings. With an aging population, increasing numbers of older adults are assuming a caregiving role and these older adult caregivers are performing complex care procedures such as wound care. The negative physical and mental health consequences of caregiving for older adult caregivers are well documented in the literature. However, access to and use of resources are associated with better physical and mental health. Past research on caregiving resources has utilized pre-determined resource variables. Little is known about older adult caregivers’ salient thoughts on resources important to caregiving and performing complex care procedures. This study utilized thematic analysis of qualitative interview data to identify themes and patterns related to resources as described by older adult caregivers. The following seven themes related to resources needed or utilized were identified: 1) expert guidance from healthcare professionals; 2) written instructions; 3) relationships with healthcare professionals for obtaining wound care supplies; 4) additional durable medical equipment; 5) financial resources; 6) coverage for caregiver personal time; and 7) select persons for social and emotional support. Older adult caregivers need and use a variety of resources when providing wound care. As increasing numbers of older adults choose to ‘age in place’, the importance of adequate resources to sustain care recipients and their caregivers in the home setting is critical.

